# Morphological and Pathogenic Variability among *Macrophomina phaseolina* Isolates Associated with Mungbean (*Vigna radiata* L.) Wilczek from Pakistan

**DOI:** 10.1155/2014/950175

**Published:** 2014-01-15

**Authors:** Umer Iqbal, Tariq Mukhtar

**Affiliations:** ^1^Crop Diseases Research Institute (DPEP), National Agricultural Research Centre, Park Road, Islamabad 45500, Pakistan; ^2^Department of Plant Pathology, Pir Mehr Ali Shah Arid Agriculture University, Rawalpindi 46300, Pakistan

## Abstract

*Macrophomina phaseolina* is a serious pathogen of many crops. In the present studies, 65 isolates of *Macrophomina phaseolina* from different agroecological regions of Punjab and Khyber Pakhtunkhwa provinces of Pakistan were analyzed for morphological and pathogenic variability. Regardless of their geographic origins, significant differences were detected among 65 isolates in their radial growth, sclerotial size, and weight as well as in pathogenicity. Sixteen isolates were rated as fast growing, 11 as slow growing, and the rest of the isolates as medium growing. Nine isolates were classified as large sized, 26 as small sized, and the remaining 30 isolates as medium sized. Thirty five isolates were ranked as heavy weight, 12 as low weight, and the rest of isolates were grouped as medium weight. Ten fungal isolates appeared to be least virulent, whereas eight isolates of diverse origin proved to be highly virulent against mungbean cultivars. The remaining isolates were regarded as moderately virulent. No relationship was found among the morphological characters and pathogenicity of the isolates. These morphological and pathogenic variations in various isolates of *M. phaseolina* may be considered important in disease management systems and will be useful in breeding programmes of mungbean cultivars resistant to charcoal rot.

## 1. Introduction

Mungbean (*Vigna radiata* L.) Wilczek is a well known summer pulse crop of Pakistan and is cultivated on an area of 245.9 thousand hectares with a total production of 177.7 thousand tones [1]. The crop is grown in a wide range of agroecological zones. The average yield of mungbean in Pakistan is very low as compared to its yield in many other countries. The low yield of mungbean in Pakistan can be attributed to legions of biotic and abiotic constraints. Among biotic factors, diseases are the most dominant. Depending upon the crop variety, the losses due to diseases to pulse crops have been estimated to be as high as 44 percent [2]. Mungbean is vulnerable to about 26 diseases in the world [3]. Among these, charcoal rot caused by *Macrophomina phaseolina* (Tassi) Goid is of prime importance in reducing crop yield especially in arid regions of the world [4]. The pathogen is distributed in diverse climatic conditions from arid to tropical regions and has a broad host range [5, 6]. There are more than 500 hosts of the fungus including legume and cereal plants [7, 8]. *M. phaseolina *is a soil- and seed- borne pathogenic fungus and produces cushion shaped black sclerotia [9]. Its prevalence can be enhanced by different physiological and ecological factors such as low moisture contents, high temperature, and heat [10, 11]. Disease severity is correlated with viable sclerotia present in the soil.

Charcoal rot infects plants at almost all growth stages. Dark lesions appear on the epicotyls and hypocotyls followed by seedling death due to obstruction of xylem vessels. In plants, the pathogen causes red to brown lesions on roots and stems with production of dark mycelia and black microsclerotia. Ultimately the plant becomes defoliated and wilted [6] and perishes [2].

Among the main management strategies, use of cultivars resistant to *M. phaseolina* has gained wide popularity and acceptance amongst farmers as application of fungicides is often intertwined with potential hazards to humans and the environment. Furthermore, resistant cultivars outstrip fungicides in various respects and emphasis is being laid on the development of new resistant germplasm. However, it has been observed that control measures against pathogens become complicated and even ineffective due to the variability among populations of the same pathogen in different agroecological zones.

There are reports in other parts of the world that populations of *M. phaseolina* showed significant variations morphologically [12], physiologically [13], pathogenically [14–17], and genetically [14–16, 18–24]. These variations aid the pathogen to adapt and survive in diverse environments.

A thorough knowledge of pathogenic variability of *M. phaseolina* is essential to design disease management strategies for different agroecological zones of the country by breeding resistant cultivars. At this time no information on the variability among *M. phaseolina* isolates is available in the country. Hence, we investigated morphological and pathogenic variability among 65 isolates of *M. phaseolina* infecting mungbean, collected from six different agroecological zones (Table 1) of Pakistan. It has also been determined whether morphological variations among *M. phaseolina* isolates have any relationship with the pathogenic variability.

## 2. Materials and Methods

### 2.1. Collection of Fungal Isolates

A total of 65 isolates of *Macrophomina phaseolina* were collected from 14 major mungbean producing districts of Punjab and Khyber Pakhtunkhwa (KPK) provinces located in six different agroecological zones of Pakistan delineated mainly on the basis of physiographic and climatic characteristics, soil type, and agricultural land use (Table 1). Samples of stems bearing microsclerotia of the fungus and characteristic symptoms of charcoal rot were collected from the infected plants from farmers' fields and research institutes and designated. The diseased samples were first packed in paper bags and then in 15 × 20 cm polyethylene bags, labelled, brought to the lab, and stored at 4°C until processed for identification.

### 2.2. Isolation, Purification, and Identification of *M. phaseolina*

The fungus was isolated from stem bark tissues of mungbean bearing fungal sclerotia and showing characteristic charcoal rot symptoms. The samples were cut into small pieces (5–10 mm long) and surface sterilized with 1% sodium hypochlorite for 2 minutes and then rinsed thrice in sterilized distilled water. The pieces were placed on Chloroneb Mercury Rose Bengal Agar (CMRA) medium [25] in Petri dishes and incubated in dark at 25 ± 1°C for 7 days. A small portion of the fastest growing colony of *M. phaseolina* was taken from the periphery of a 90 mm diameter Petri dish, spread onto Petri dishes containing glucose agar medium (glucose, 20 g; agar, 20 g; and water, 1 L), and incubated in the dark at 25 ± 1°C for 7 days. A small portion of the colony having sclerotia was taken up into a drop of sterilized water and agitated with a sterilized needle to separate the sclerotia from the mycelia. Sclerotia were then transferred to 90 mm diameter Petri dishes containing CMRA medium. Colonies appearing from single sclerotium were again transferred to CMRA medium in 90 mm Petri plates, incubated as mentioned above and identified as described [26].

### 2.3. Storage of Pure Cultures of *M. phaseolina*

The purified culture (5 mm disc) from each isolate growing on PDA was transferred to 10 mL culture tubes and incubated in the dark at 25 ± 1°C for 6 days, until the surface of PDA was covered with a dense sclerotial layer of the fungal culture. The culture tubes were labeled and stored at 4°C.

### 2.4. Multiplication of *M. phaseolina*

Ground sorghum seeds were water-soaked overnight, air-dried under room temperature, and placed in conical flasks. The mouth of each flask was plugged with cotton wool, wrapped in aluminum foil, and autoclaved at 15 psi (121°C) for 20 minutes. After cooling, the seeds in flasks were inoculated with 4 mm mycelial plugs from a 7-day old culture of *M. phaseolina *and incubated at 25 ± 1°C for 15 days. The flasks were shaken at alternate days for uniform colonization of the grains. The inoculum thus produced was used in pot assay.

### 2.5. Determination of Morphological Variability

Morphological variability among 65 isolates of *M. phaseolina* was studied on the basis of the following parameters.

#### 2.5.1. Radial Growth

For studying variability in radial growth, the isolates were grown on Potato Dextrose Agar [25]. Fifteen milliliters of autoclaved PDA was poured in 90 mm diameter Petri plates, allowed to solidify, and inoculated in the center with a 5 mm plug from the actively growing culture of each isolate of the fungus. The plates were incubated at 25 ± 1°C for 7 days. Each isolate was replicated five times. After the stipulated period, the growth of each isolate was measured in terms of colony diameter and their means were computed. On the basis of radial growth, the isolates were categorized as fast (>80 mm), medium (61–80 mm), and slow (<61 mm) growing.

#### 2.5.2. Sclerotial Size

For measuring sclerotial size, slides from 7-day-old pure cultures of *M. phaseolina* isolates were prepared and examined under a microscope ocular micrometer. Sizes of ten randomly selected sclerotia were measured and their means were calculated. The isolates were classified as large (>25 *μ*m), medium (21–25 *μ*m), and small (<21 *μ*m) sized.

#### 2.5.3. Sclerotial Weight

In order to measure the dry weight of sclerotia, each isolate of the fungus was cultured in 100 mL sterilized Potato Dextrose Broth in 250 mL Erlenmeyer flasks with five replicates. The flasks were incubated at 25 ± 1°C for 15 days. The sclerotia were filtered through Whatman filter paper no. 41, wrapped in aluminium foil, and oven-dried at 45°C for 24 hrs. The sclerotia of each isolate were weighed using electric balance and grouped as heavy (>0.15 mg), medium (0.11–0.15 mg), and low (<0.11 mg) weight.

### 2.6. Determination of Pathogenic Variability

The pathogenicity of 65 isolates was studied on three cultivars of mungbean (NM-92, NM-51, and AEM-96) in the glasshouse in a split plot design with cultivars as main plots and the isolates as subplots. Each treatment was repeated thrice. Seeds were disinfected by immersing in 2.5% NaOCl for 5 min, rinsed in sterilized water, and air-dried.

Ten seeds of each of the three cultivars of mungbean were sown in pots containing 2 kg soil infested with each isolate of *M. phaseolina* @ 2 g/kg soil. Pots without inoculum served as controls. The pots were placed in a glasshouse at 30 ± 2°C. Disease severity caused by each isolate on each cultivar was assessed after 20 days of emergence using the disease rating scale developed by Abawi and Pastor-Corrales [6].

### 2.7. Statistical Analysis

Data were subjected to analysis of variance (ANOVA) using GenStat package 2009 (12th edition) version 12.1.0.3278 (http://www.vsni.co.uk). The differences among means were compared by Fisher's protected least significant difference test at *P* ≤ 0.05. Euclidean distances were used to construct a dendrogram by unweighted paired group method with arithmetic averages (UPGMA) using Statistica version 6.1.

## 3. Results

### 3.1. Morphological Variability among *M. phaseolina* Isolates

Significant variations were observed in the morphological parameters among 65 isolates of *M. phaseolina* collected from six agroecological zones of Pakistan.

#### 3.1.1. Radial Growth

Significant differences among 65 isolates of *M. phaseolina* collected from different districts were observed on the basis of radial growth (*F* = 11.75; df = 64, 130; *P* < 0.001). The individual average radial growths of 65 isolates of *M. phaseolina* ranged from 32.00 to 87.17 mm observed 7 days after incubation. Maximum colony diameters of 87.17 and 86.67 mm were observed in case of isolate MP-7 (Dera Ghazi Khan) and MP-26 (Layyah) proving to be the fast growing, while isolates MP-8, MP-29, and MP-30 showed the minimum radial growths and were rated as slow growing. The individual radial growths of all the isolates are shown in Table 2 (Column 2). Sixteen isolates showed radial growths above 80 mm and were rated as fast growing while the growth of 11 isolates was found below 61 mm and were categorized as slow growing. The rest of the isolates showed growth between 61 and 80 mm and hence were classified as medium growing (Table 3).

#### 3.1.2. Sclerotial Size

Significant variations were also observed among these isolates regarding the size of their sclerotia (*F* = 3.53; df = 64, 130; *P* < 0.001). Maximum sclerotial size was observed in case of isolates MP-20 and MP-3 showing 29.00 and 27.33 *μ*m diameter, respectively, while the isolates MP-39 and MP-28 were found to be the smallest in size. The individual average sclerotial sizes of isolates ranged from 17.00 to 29.00 *μ*m which are given in Table 2 (Column 3). The size of sclerotia of 9 isolates was above 25 *μ*m and were classified as large size while 26 isolates with sclerotial size less than 21 *μ*m were rated as small sized. The remaining 30 isolates ranged between 21 and 25 *μ*m in sclerotial size and were categorized as medium sized (Table 4).

#### 3.1.3. Sclerotial Weight

Sclerotial weight was another parameter considered for variability. The analysis of variance showed significant variability in sclerotial weight among the isolates (*F* = 6.07; df = 64, 130; *P* < 0.001). Analyzing the data of sclerotial weight revealed that isolates MP-20, MP-23, MP-24, and MP-52 produced maximum sclerotia giving maximum weight above 0.20 mg. The lowest sclerotial production was found in isolate MP-46 with average weight of 0.08 mg. The mean individual sclerotial weight of each isolate is given in Table 2 (Column 4). Thirty five isolates were ranked as heavy weight as these produced sclerotia more than 15 mg while 12 isolates produced sclerotia weighing less than 11 mg and were graded as low weight. The rest of isolates being weighed between 11 to 15 mg were grouped as medium weight (Table 5).

#### 3.1.4. Cluster Analysis Based on Radial Growth, Sclerotial Size and Sclerotial Weight

**Figure 1:**
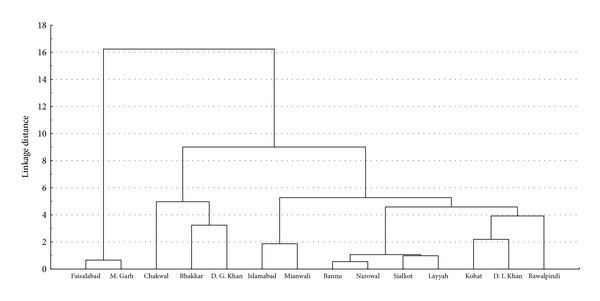
Dendrogram derived from cluster analysis (UPGMA) showing relationship among the 65 isolates of *M. phaseolina* on the basis of morphological characters collected from 14 districts of Punjab and KPK provinces.

The cluster analysis of 65 isolates of 14 districts on an average basis of three morphological parameters (radial growth, sclerotial size, and sclerotial weight) is shown in Figure 1. In the dendrogram, three main clusters were distinguished, at a linkage distance of around 50%. The first cluster comprised 8 isolates of Faisalabad and Muzaffar Garh districts; the second cluster comprised 13 isolates of Chakwal, Bhakkar, and Dera Ghazi Khan districts, and the third cluster consisted of 44 isolates of the remaining districts. The isolates belonging to the districts Chakwal, Bhakkar, and D.G. Khan showed optimum growth performance, while the isolates belonging to M. Garh and Faisalabad exhibited poor growth performance. The isolates in the third group were found intermediate in their growth performance.

### 3.2. Pathogenic Variability among *M. phaseolina* Isolates

Highly significant differences were observed among isolates, varieties, and their interactions. Significant variations in pathogenicity were found among 65 isolates of the fungus (*F* = 34.31; df = 64, 388; *P* < 0.001) when tested against three mungbean cultivars which also varied in response to the isolates (*F* = 52.049; df = 2, 388; *P* < 0.001). Six isolates, namely, MP-7, MP-13, MP-18, MP-48, MP-56, and MP-64, were found highly virulent against NM-92 with mean disease severity scoring of 7.3. Five isolates, namely, MP-8, MP-10, MP-26, MP-35, and MP-60, were found to be the least virulent in their reaction with average disease score ranging between 2.3 and 3.7 showing that the cultivar is resistant against these isolates (Table 6, Column 2).

Similarly nine isolates, namely, MP-5, MP-16, MP-17, MP-22, MP-33, MP-37, MP-44, MP-56, and MP-63, were detected to be virulent against NM-51 with an average disease score ranging from 7.0 to 7.7, while six isolates, namely, MP-11, MP-30, MP-31, MP-38, MP-53, and MP-60, exhibited least pathogenic reaction against NM-51 with disease severity ranging from 2.0 to 3.0 and the remaining isolates proved to be intermediate in their pathogenicity (Table 6, Column 3).

Seventeen isolates appeared to be highly virulent towards AEM-96 as these gave disease scores above 7, while six isolates, namely, MP-8, MP-25, MP-30, MP-31, MP-35, MP-36, and MP-41, with an average disease score ranged up to 3 proved to be least virulent. The rest of the isolates were found to be moderately virulent (Table 6, Column 4).

**Figure 2:**
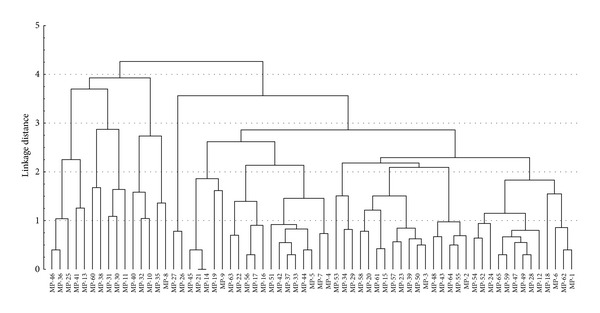
Dendrogram showing the clustering of the virulence of *M. phaseolina* isolates on 3 mungbean cultivars.

The cluster analysis on the basis of pathogenicity is shown in Figure 2. Sixty five isolates were categorized into five clusters on the basis of pathogenicity against three mungbean cultivars. Ten fungal isolates placed in clusters 2 and 3 appeared to be least virulent, whereas eight isolates of diverse origin in cluster 5 proved to be highly virulent for their virulence against mungbean cultivars. The remaining isolates falling under clusters 1 and 4 were regarded as moderately virulent.

## 4. Discussion

*Macrophomina phaseolina*, a soil- as well as seed-borne fungus, induces charcoal rot in different crops including mungbean. In the present studies, 65 isolates of *M. phaseolina* belonging to different regions of Punjab and Khyber Pakhtunkhwa provinces of Pakistan showed variations in different morphological traits such as radial growth, sclerotial size, and weight as well as in pathogenicity. The variations in morphology might be due to differences in temperature, moisture, soil types, and other edaphic factors of various districts of Punjab and KPK. Morphological variability has also been reported by many workers in terms of growth, color, pycnidium production, and chlorate sensitivity among different isolates of *M. phaseolina* on different hosts [11, 27–31] which corroborated our findings. Similarly, variations in morphology and pathogenicity among *M. phaseolina* isolates taken from different hosts as well as from different parts of the same host have also been observed by Beas-Fern$\overset{´}{\text{a}}$ndez et al. [32]. However, in the present studies, no relationship was found among the morphological characters and pathogenicity of the isolates. Among the highly virulent isolates of *M. phaseolina*,namely, MP-7, MP-13, MP-18, MP-48, MP-56, and MP-64, against mungbean, not all the isolates were fast growing (radial growth > 80 mm) or large sized (>25 *μ*m) or have high weight (>0.15 mg). Of these highly virulent isolates, two (MP-7 and MP-48) were the fast growing, and the remaining 4 were medium growing. Similarly, isolates MP-18 and MP-26 produced large sized, MP-7 and MP-13 medium sized, and MP-48 and MP-64 small sized sclerotia. Likewise, MP-7, MP-13, MP-18, and MP-64 were high and MP-48 was low weight. Similar pattern was observed in moderately and least virulent isolates. Confirmatory and contradictory findings in this regard have also been reported by others. A close linkage between virulence and growth was reported by Rayner [33]. Purkayastha et al. [34] also found relationship between morphological variations and pathogenicity. On the other hand, Dhingra and Sinclair [11] and Beas-Fernández et al. [32] reported that pathogenicity has no relation with size and weight of sclerotia.

The pathogenic fungus, *M. phaseolina,* has a broad host range and exits in two asexual forms which maintain its survival better [11, 13, 35]. Some workers also related variability to the phenomena of host specialization in *M. phaseolina*. Su et al. [15] found host specialization in maize on the basis of pathogenic, genetic, and physiological differences. Similarly, Cloud and Rupe [35] analyzed host specialization in soybean. This mechanism takes long time to establish within a specific host. Mihail and Taylor [13] suggested that, due to heterogenic nature of *M. phaseolina*, categorization into distinct subgroups based upon pathogenicity and morphology could not take place. Pathogenesis along with genetic diversity plays a specific role in host-plant resistance. Isolates having morphological similarity are not necessarily identical genetically, they might have some differences. The variable genetic pattern contributes to variation in morphology and pathogenesis, which has been confirmed by using different molecular tools [14, 16, 21, 22, 36–38]. As the pathogen has no sexual phase, genetic diversity is produced either by fusion of vegetative cells or by parasexual recombination between nuclear genes [39]. In nature genetic variability improves survival of a fungus [37].

It is quite evident that variability in morphology, physiology, genetics, pathogenicity, and so forth is imperative for the fungus to have better adaptation in response to diversified environmental behavior. It also leads to host-plant resistance, development of resistant varieties of different crops against disease, and implementation of new disease controlling strategies [14, 40].

## 5. Conclusions

The determination of variability among *M. phaseolina* isolates is fundamental to guide the development of appropriate strategies for disease management according to different agroecological zones. As there are no reports about the determination of morphological and pathogenic variability, the present studies for the first time provide information on the variability of *M. phaseolina* in major mungbean growing areas of Pakistan. These results will be useful in developing integrated strategies for the management of charcoal rot and breeding programs for pulses and other crops.

## Figures and Tables

**Table 1:** Isolates of *Macrophomina phaseolina *collected from mungbean plants from different agroecological zones.

Agroecological Zone	Soil type	District	Isolate
Sandy desert	Sandy and loamy fine sandy soil, moderately to strongly calcareous	Mianwali	MP-12, MP-13, MP-14, MP-15, MP-16
		Bhakkar	MP-17, MP-18, MP-19, MP-20, MP-21
		Layyah	MP-22, MP-23, MP-24, MP-25, MP-26, MP-27, MP-28

Northern irrigated plain	Sandy loam to clay loam	Faisalabad	MP-29, MP-30, MP-31, MP-32, MP-33
		Muzaffargarh	MP-8, MP-9, MP-10, MP-11

Barani (rainfed)	Non-calcareous to moderately calcareous silt loams	Narowal	MP-34, MP-35, MP-36
		Sialkot	MP-37, MP-38, MP-39, MP-40, MP-41
		Chakwal	MP-42, MP-43, MP-44, MP-45

Wet mountains	Silt loam to silty clays, non-calcareous to slightly calcareous	Islamabad	MP-46, MP-47, MP-48, MP-49
		Rawalpindi	MP-1, MP-2, MP-3

Western dry mountains	Loamy, deep, and strongly calcareous	Kohat	MP-56, MP-57, MP-58, MP-59
		Banu	MP-60, MP-61, MP-62, MP-63, MP-64, MP-65

Sulaiman piedmont	Loamy to clayey and strongly calcareous	Dera Ghazi Khan	MP-4, MP-5, MP-6, MP-7
		Dera Ismail Khan	MP-50, MP-51, MP-52, MP-53, MP-54, MP-55

**Table 2:** Morphological variations among different isolates of *M. phaseolina *

Isolates	Radial growth (mm)	Sclerotial size (*μ*m)	Sclerotial weight (mg)
1	2	3	4
MP-1	63.17 i-r	26.67 a-d	0.10 h-l
MP-2	65.33 i-p	23.50 b-l	0.12 e-l
MP-3	75.17 a-i	27.33 ab	0.10 h-l
MP-4	71.67 f-m	19.67 g-o	0.13 d-l
MP-5	72.33 e-m	20.67 f-o	0.15 a-k
MP-6	78.00 a-h	20.33 f-o	0.16 a-h
MP-7	87.17 a	24.17 a-j	0.15 a-k
MP-8	32.00 t	20.83 f-o	0.11 g-l
MP-9	52.83 q-s	24.67 a-h	0.13 d-l
MP-10	83.00 a-f	23.00 b-m	0.13 d-l
MP-11	54.67 o-s	19.67 g-o	0.15 a-k
MP-12	64.33 i-r	18.17 l-o	0.16 a-h
MP-13	68.33 h-n	21.17 e-o	0.15 a-k
MP-14	84.67 a-e	19.33 h-o	0.13 d-l
MP-15	74.50 b-k	19.00 i-o	0.14 c-l
MP-16	68.00 h-n	21.83 c-o	0.14 b-l
MP-17	82.17 a-g	24.50 a-i	0.18 a-f
MP-18	74.83 a-j	26.83 a-c	0.19 a-d
MP-19	85.33 a-c	21.17 e-o	0.19 a-d
MP-20	62.50 j-r	29.00 a	0.21 a
MP-21	78.00 a-h	20.33 f-o	0.17 a-g
MP-22	71.83 f-m	20.17 f-o	0.19 a-d
MP-23	65.17 i-q	17.50 m-o	0.20 ab
MP-24	64.00 i-r	22.50 b-o	0.20 a-c
MP-25	66.33 h-o	22.67 b-n	0.18 a-f
MP-26	86.67 ab	23.33 b-l	0.17 a-g
MP-27	82.83 a-f	21.67 c-o	0.18 a-f
MP-28	43.83 s	17.33 no	0.17 a-g
MP-29	43.67 s	18.00 l-o	0.13 d-l
MP-30	44.00 s	22.67 b-n	0.13 d-l
MP-31	53.83 p-s	26.33 a-e	0.15 a-k
MP-32	83.33 a-f	19.83 g-o	0.12 f-l
MP-33	84.17 a-f	17.67 m-o	0.12 f-l
MP-34	53.17 p-s	21.83 c-o	0.10 h-l
MP-35	72.50 d-m	24.67 a-h	0.13 d-l
MP-36	61.50 m-r	22.17 b-o	0.10 h-l
MP-37	52.33 rs	19.33 h-o	0.13 d-l
MP-38	73.00 c-m	17.50 m-o	0.09 kl
MP-39	67.33 h-n	17.00 o	0.09 i-l
MP-40	74.17 b-l	25.00 a-g	0.10 h-l
MP-41	71.83 f-m	23.50 b-l	0.12 e-l
MP-42	73.17 c-m	24.33 a-j	0.13 d-l
MP-43	81.33 a-g	18.83 j-o	0.12 e-l
MP-44	85.00 a-d	19.00 i-o	0.12 e-l
MP-45	86.17 ab	22.50 b-o	0.13 d-l
MP-46	84.83 a-e	17.50 m-o	0.08 l
MP-47	65.17 i-q	21.33 d-o	0.09 kl
MP-48	81.17 a-g	18.50 k-o	0.10 h-l
MP-49	64.17 i-r	22.33 b-o	0.09 j-l
MP-50	81.83 a-g	20.33 f-o	0.18 a-f
MP-51	56.50 n-r	21.67 c-o	0.17 a-g
MP-52	78.00 a-h	25.67 a-f	0.20 ab
MP-53	67.17 h-n	23.50 b-l	0.18 a-f
MP-54	78.50 a-h	23.50 b-l	0.19 a-d
MP-55	63.83 i-r	24.17 a-j	0.20 a-c
MP-56	62.17 k-r	27.50 ab	0.16 a-i
MP-57	71.83 f-m	22.83 b-n	0.18 a-e
MP-58	74.17 b-l	24.00 a-k	0.15 a-k
MP-59	78.17 a-h	26.67 a-d	0.16 a-i
MP-60	61.17 m-r	19.33 h-o	0.18 a-e
MP-61	69.67 g-m	19.67 g-o	0.18 a-f
MP-62	84.50 a-e	21.50 c-o	0.17 a-g
MP-63	61.67 l-r	24.17 a-j	0.15 a-k
MP-64	78.67 a-h	19.83 g-o	0.15 a-j
MP-65	54.50 o-s	24.17 a-j	0.16 a-i

Values are means of five replicates in case of radial growth and sclerotial weight and ten replicates in case of sclerotial size.

Values sharing common letters in each column do not differ significantly at *P* < 0.05 according to Fisher's protected least significant difference test.

**Table 3:** Categorization of *M. phaseolina* isolates on the basis of radial growth.

S. No.	Category	Number	Isolates
1	Fast growing (>80 mm)	16	MP-7, MP-10, MP-14, MP-17, MP-19, MP-26, MP-27, MP-32, MP-33, MP-43, MP-44, MP-45, MP-46, MP-48, MP-50, MP-62

2	Medium growing (61–80 mm)	38	MP-1, MP-2, MP-3, MP-4, MP-5, MP-6, MP-12, MP-13, MP-15, MP-16, MP-18, MP-20, MP-21, MP-22, MP-23, MP-24, MP-25, MP-35, MP-36, MP-38, MP-39, MP-40, MP-41, MP-42, MP-47, MP-49, MP-52, MP-53, MP-54, MP-55, MP-56, MP-57, MP-58, MP-59, MP-60, MP-61, MP-63, MP-64

3	Slow growing (<61 mm)	11	MP-8, MP-9, MP-11, MP-28, MP-29, MP-30, MP-31, MP-34, MP-37, MP-51, MP-65

**Table 4:** Categorization of *M. phaseolina* isolates on the basis of size of sclerotia.

S. No.	Category	Number	Isolates
1	Large sized (>25 *µ*m)	9	MP-1, MP-3, MP-18, MP-20, MP-31, MP-40, MP-52, MP-56, MP-59

2	Medium sized (21–25 *µ*m)	30	MP-2, MP-7, MP-9, MP-10, MP-13, MP-16, MP-17, MP-19, MP-24, MP-25, MP-26, MP-27, MP-30, MP-34, MP-35, MP-36, MP-41, MP-42, MP-45, MP-47, MP-49, MP-51, MP-53, MP-54, MP-55, MP-57, MP-58, MP-62, MP-63, MP-65

3	Small sized (<21 *µ*m)	26	MP-4, MP-5, MP-6, MP-8, MP-11, MP-12, MP-14, MP-15, MP-21, MP-22, MP-23, MP-28, MP-29, MP-32, MP-33, MP-37, MP-38, MP-39, MP-43, MP-44, MP-46, MP-48, MP-50, MP-60, MP-61, MP-64

**Table 5:** Categorization of *M. phaseolina* isolates on the basis of weight of sclerotia

S. No.	Category	Number	Isolates
1	Heavy weight (>0.15 mg)	35	MP-5, MP-6, MP-7, MP-11, MP-12, MP-13,MP-17, MP-18, MP-19, MP-20, MP-21, MP-22, MP-23, MP-24, MP-25, MP-26, MP-27, MP-28, MP-31, MP-50, MP-51, MP-52, MP-53, MP-54, MP-55, MP-56, MP-57, MP-58, MP-59, MP-60, MP-61, MP-62, MP-63, MP-64, MP-65

2	Medium weight (0.11–0.15 mg)	18	MP-2, MP-4, MP-9, MP-10, MP-14, MP-15, MP-16, MP-29, MP-30, MP-32, MP-33, MP-35, MP-37, MP-41, MP-42, MP-43, MP-44, MP-45

3	Low weight (<0.11 mg)	12	MP-1, MP-3, MP-8, MP-34, MP-36, MP-38, MP-39, MP-40, MP-46, MP-47, MP-48, MP-49

**Table 6:** Differential response of selected mungbean cultivars against various isolates of *M. phaseolina*.

Isolates	NM-92	NM-51	AEM-96
1	2	3	4
MP-1	5.7 defg	5.7 defg	5.7 defg
MP-2	7.0 abc	4.7 ghij	7.3 ab
MP-3	5.7 defg	5.3 efgh	7.0 abc
MP-4	6.7 abcd	7.0 abc	7.3 ab
MP-5	6.0 cdef	7.7 a	7.3 ab
MP-6	6.3 bcde	5.7 defg	6.0 cdef
MP-7	7.3 ab	6.7 abcd	7.0 abc
MP-8	2.7 mn	5.7 defg	3.0 lmn
MP-9	4.3 hijk	5.3 efgh	4.7 ghij
MP-10	2.3 mn	5.7 defg	5.0 fghi
MP-11	5.7 defg	2.7 mn	4.3 hijk
MP-12	6.0 cdef	4.7 ghij	5.7 defg
MP-13	7.3 ab	6.0 cdef	4.3 hijk
MP-14	4.7 ghij	7.0 abc	5.3 efgh
MP-15	4.3 hijk	5.0 fghi	7.0 abc
MP-16	6.3 bcde	7.3 ab	5.7 defg
MP-17	7.0 abc	7.3 ab	6.0 cdef
MP-18	7.3 ab	5.7 defg	5.7 defg
MP-19	5.7 defg	6.0 cdef	4.3 hijk
MP-20	4.3 hijk	6.3 bcde	7.3 ab
MP-21	4.7 ghij	7.0 abc	5.3 efgh
MP-22	6.0 cdef	7.3 ab	4.7 ghij
MP-23	5.3 efgh	5.7 defg	7.7 a
MP-24	4.7 ghij	5.0 fghi	5.7 defg
MP-25	5.7 defg	5.3 efgh	2.7 mn
MP-26	2.7 mn	6.7 abcd	7.3 ab
MP-27	3.3 klm	7.0 abc	7.7 a
MP-28	5.3 efgh	4.3 hijk	5.7 defg
MP-29	6.0 cdef	4.0 ijk	7.0 abc
MP-30	5.3 efgh	2.3 mn	3.0 lmn
MP-31	4.3 hijk	2.0 n	3.3 klm
MP-32	2.3 mn	4.7 ghij	4.7 ghij
MP-33	5.7 defg	7.3 ab	6.7 abcd
MP-34	5.3 efgh	4.3 hijk	7.3 ab
MP-35	3.3 klm	4.7 ghij	2.3 mn
MP-36	5.3 efgh	4.3 hijk	2.7 mn
MP-37	5.7 defg	7.3 ab	7.0 abc
MP-38	4.3 hijk	3.0 lmn	5.3 efgh
MP-39	6.0 cdef	5.7 defg	6.7 abcd
MP-40	2.7 mn	4.0 ijk	5.7 defg
MP-41	7.0 abc	5.3 efgh	3.3 klm
MP-42	5.7 defg	7.0 abc	7.3 ab
MP-43	6.7 abcd	5.7 defg	7.0 abc
MP-44	6.0 cdef	7.7 a	7.7 a
MP-45	4.7 ghij	7.0 abc	5.7 defg
MP-46	5.7 defg	4.3 hijk	2.7 mn
MP-47	5.3 efgh	4.7 ghij	5.7 defg
MP-48	7.3 ab	6.0 cdef	7.0 abc
MP-49	5.3 efgh	4.0 ijk	5.7 defg
MP-50	5.7 defg	5.7 defg	7.3 ab
MP-51	5.3 efgh	7.0 abc	7.7 a
MP-52	4.7 ghij	4.3 hijk	5.3 efgh
MP-53	5.7 defg	2.7 mn	7.3 ab
MP-54	4.3 hijk	4.0 ijk	5.7 defg
MP-55	7.0 abc	5.3 efgh	7.3 ab
MP-56	7.3 ab	7.3 ab	6.0 cdef
MP-57	5.7 defg	5.3 efgh	7.7 a
MP-58	4.7 ghij	5.7 defg	7.0 abc
MP-59	5.7 defg	4.3 hijk	5.3 efgh
MP-60	3.7 jkl	2.3 mn	6.7 abcd
MP-61	4.3 hijk	4.7 ghij	7.3 ab
MP-62	5.3 efgh	5.7 defg	5.7 defg
MP-63	6.7 abcd	7.3 ab	4.7 ghij
MP-64	7.3 ab	5.3 efgh	7.7 a
MP-65	5.7 defg	4.0 ijk	5.3 efgh

Values are means of three replicates.

Values sharing common letters in each column do not differ significantly at *P* < 0.05 according to Fisher's protected least significant difference test.
